# Post-training Load-Related Changes of Auditory Working Memory – An EEG Study

**DOI:** 10.3389/fnhum.2020.00072

**Published:** 2020-03-17

**Authors:** Helene Gudi-Mindermann, Johanna M. Rimmele, Patrick Bruns, Niels A. Kloosterman, Tobias H. Donner, Andreas K. Engel, Brigitte Röder

**Affiliations:** ^1^Department of Biological Psychology and Neuropsychology, University of Hamburg, Hamburg, Germany; ^2^Department of Neurophysiology and Pathophysiology, University Medical Center Hamburg-Eppendorf, Hamburg, Germany; ^3^Department of Neuroscience, Max-Planck-Institute for Empirical Aesthetics, Frankfurt am Main, Germany; ^4^Max Planck UCL Centre for Computational Psychiatry and Ageing Research, Max-Planck-Institute for Human Development, Berlin, Germany

**Keywords:** auditory working memory, working memory load, post-training plasticity, EEG, source space

## Abstract

Working memory (WM) refers to the temporary retention and manipulation of information, and its capacity is highly susceptible to training. Yet, the neural mechanisms that allow for increased performance under demanding conditions are not fully understood. We expected that post-training efficiency in WM performance modulates neural processing during high load tasks. We tested this hypothesis, using electroencephalography (EEG) (*N* = 39), by comparing source space spectral power of healthy adults performing low and high load auditory WM tasks. Prior to the assessment, participants either underwent a modality-specific *auditory* WM training, or a modality-irrelevant *tactile* WM training, or were not trained (active control). After a modality-specific training participants showed higher behavioral performance, compared to the control. EEG data analysis revealed general effects of WM load, across all training groups, in the theta-, alpha-, and beta-frequency bands. With increased load theta-band power increased over frontal, and decreased over parietal areas. Centro-parietal alpha-band power and central beta-band power decreased with load. Interestingly, in the high load condition a tendency toward reduced beta-band power in the right medial temporal lobe was observed in the modality-specific WM training group compared to the modality-irrelevant and active control groups. Our finding that WM processing during the high load condition changed after modality-specific WM training, showing reduced beta-band activity in voice-selective regions, possibly indicates a more efficient maintenance of task-relevant stimuli. The general load effects suggest that WM performance at high load demands involves complementary mechanisms, combining a strengthening of task-relevant and a suppression of task-irrelevant processing.

## Introduction

Working memory (WM) has been defined as the ability to temporary maintain and manipulate stored information (Baddeley, 2003; D’Esposito, 2007; Jonides et al., 2008). Language processing highly relies on WM processes, as information needs to be maintained and integrated over time, for example during phrasal or sentence level processing (Montgomery, 2000; Emmorey et al., 2017). Particularly verbal WM is crucial for speech comprehension (Buchsbaum and D’Esposito, 2019), but speech comprehension additionally requires the processing of extralinguistic cues, such as voice features and prosody (Larrouy-Maestri et al., 2013; Hellbernd and Sammler, 2016). Language learning can benefit from prosodic cues, suggesting interactions of verbal and extralinguistic memory (Schon et al., 2008; de Diego-Balaguer et al., 2015). Here, in a voice recognition task, we focus on auditory WM of extralinguistic cues. WM capacity varies among individuals (Luck and Vogel, 2013), but can be improved by training (Morrison and Chein, 2011), such that tasks of higher difficulty can be managed successfully following training. The present study investigated the neural mechanisms that allow enhanced auditory WM performance at high difficulty levels following WM training.

**FIGURE 1:**
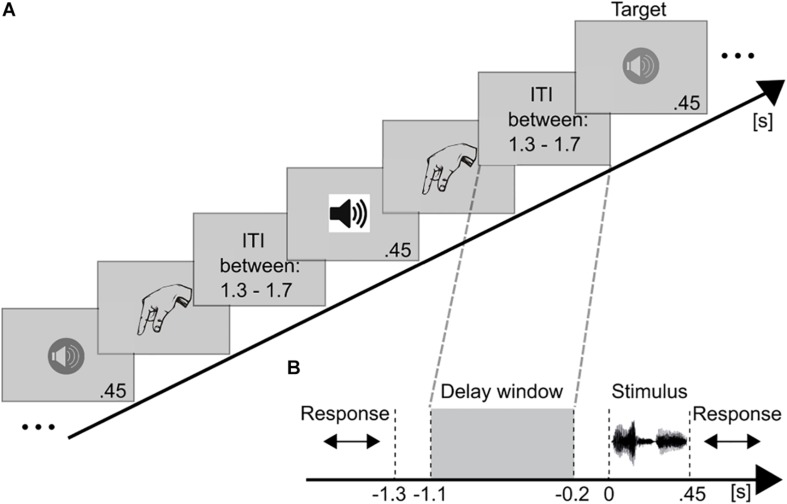
Schematic illustration of the paradigm and trial structure. **(A)** Example for a stimulus sequence in the auditory 2-back task. Auditory voice stimuli were successively presented. Different symbols for the loudspeakers represent different voices. After each stimulus the participant indicated via button press whether (target) or not (non-target) the current speaker matched the speaker presented *n* = 2 trials earlier. After an inter-trial-interval (ITI) the next stimulus was presented. **(B)** The temporal course of a trial is presented time-locked to stimulus onset (0 s). After each stimulus (450 ms) a response was required (unlimited), followed by the ITI (randomized between 1.3 and 1.7 s). Thus, the shortest working memory maintenance period (highlighted by gray dashed lines) lasted for 1.3 s. This period was used as the time window of interest. EEG data of this maintenance period (–1.1 to –0.2 s) were analyzed.

A classical paradigm to assess WM processing at several difficulty levels is the *n*-back task. In *n*-back tasks participants receive a stimulus sequence and have to decide whether or not the current stimulus matches the stimulus presented *n* trials before (Figure 1A). The *n*, thus, represents the adjustable load factor; the higher the *n*, the higher the WM demands. Electroencephalography (EEG) and magnetoencephalography (MEG) studies have reported a parametric relationship between increasing WM load and oscillatory activity (typically neuronal power increases), mainly in the theta- (Krause et al., 2000; Jensen and Tesche, 2002; Hellbernd and Sammler, 2016) and gamma-bands (Kaiser et al., 2003; Palva et al., 2011; Roux et al., 2012). Furthermore, this WM-related theta- and gamma-band activity has been predominantly associated with frontal (Jensen and Tesche, 2002; Barnett et al., 2008; Dupoux et al., 2008; Kaiser et al., 2009) and parietal areas (Sauseng et al., 2009; Scharinger et al., 2017; Kapeller et al., 2018), which are commonly considered to represent the core WM network (for reviews see Wager and Smith, 2003; D’Esposito and Postle, 2015; Eriksson et al., 2015). In WM tasks, the fronto-parietal theta- and gamma-band oscillatory activity have been suggested to reflect retention of relevant information (for a review see Roux and Uhlhaas, 2014).

In contrast, the functional roles of alpha- and beta-band activity in WM have not been yet clearly defined (for a review see Roux and Uhlhaas, 2014). For example, alpha-band modulations have been consistently reported to vary with WM load. Nevertheless, the direction of the relationship remains controversial, reporting both load-induced alpha-band increases (Scheeringa et al., 2009; van Dijk et al., 2010) and load-induced alpha-band decreases (Haegens et al., 2014; Chen and Huang, 2015). A positive relation between WM load and synchronized alpha power (Jokisch and Jensen, 2007; Medendorp et al., 2007) has been interpreted in the light of the inhibition-timing hypothesis (Klimesch et al., 2007), which has linked increases in alpha-band amplitude with an inhibition of task-irrelevant brain regions. Conversely, a decrease in alpha-band amplitude has been associated with a release from inhibition (Klimesch et al., 2007; Klimesch, 2012) and an overall enhanced cortical engagement at higher load demands (Gevins et al., 1997; Palomaki et al., 2012; Chen and Huang, 2015). Similarly, the contribution of beta-band power to WM is not well understood. Although several studies have reported beta-band activity to be modulated by WM load (Deiber et al., 2007; Chen and Huang, 2015; Palva et al., 2011; Scharinger et al., 2017), beta-band power has not been particularly associated with a specific functional role in WM tasks (for reviews see Roux and Uhlhaas, 2014; D’Esposito and Postle, 2015; Eriksson et al., 2015). Some studies, however, propose a role for (low) beta-band activity in short-term memory (Kopell et al., 2011), cognitive WM processes (Scharinger et al., 2017) or more general integrative functions (Donner and Siegel, 2011) such as the maintenance of the current (motor or cognitive) state, as it is required during WM delay periods (Engel and Fries, 2010). Furthermore, functional connectivity particularly in the beta-band has been found to be enhanced between WM-relevant frontal and parietal regions in WM tasks (Palva et al., 2010, 2011; Salazar et al., 2012).

WM training has been shown to alter oscillatory activity in WM-relevant regions. For example, behavioral training gains in WM tasks were accompanied by training-induced increases in frontal (Gevins et al., 1997; Langer et al., 2013) and fronto-parietal (Jausovec and Jausovec, 2012) theta-band power, suggesting that training strengthened the WM network, thereby, facilitating WM performance. Furthermore, functional connectivity has been found to be altered by WM training (e.g., Langer et al., 2013; Astle et al., 2015; Gudi-Mindermann et al., 2018; Rimmele et al., 2019). While power increases are thought to reflect local processing (Donner and Siegel, 2011; Buzsaki and Wang, 2012), functional connectivity is assumed to reflect the degree of the temporal alignment of brain activity in distributed networks (Engel and Singer, 2001; Varela et al., 2001; Womelsdorf et al., 2007). A training study in children reported that 20–25 sessions of a computerized verbal and spatial WM training relative to a control group enhanced the coupling of resting state MEG activity between a fronto-parietal network and lateralized occipital and inferior temporal regions (Astle et al., 2015). In a consecutive investigation of the same children, the authors reported training-induced increases in cross-frequency phase amplitude coupling (Barnes et al., 2016): Following training, gamma-band power (∼90 Hz) in inferior-parietal and temporal areas was phase-locked to a slower beta rhythm (16 Hz) at fronto-parietal areas. These findings demonstrate that neural mechanisms involved in WM processing change as a function of training, as indicated by training-induced changes in both oscillatory power and functional connectivity. Typically, such changes are assessed by contrasting EEG activity prior to and after training while the same task is performed. Therefore, it remains unclear how WM processing at different load demands is affected by the training-induced neuronal changes. While trained and un-trained individuals might perform similar at low load demands, the question arises how the neurophysiological training-induced changes facilitate WM processing at high load demands, i.e., how the underlying mechanisms are altered in trained relative to non-trained individuals.

The present study investigated how post-training performance proficiency affects the neural mechanisms involved in successful WM processing at high load demands. Healthy adults performed a low load (2-back) and a high load (adaptive *n*-back) auditory WM task with voices. In the high load condition, the *n*-back level was continuously adjusted to the participants performance, such that participants were continuously performing at their capacity limits. This ensured that participants, despite interindividual differences in WM capacity, always performed at high load demands. Prior to the test session, participants were adaptively trained (adaptive *n*-back) either with the same auditory voice stimuli as in the test session (auditory training group), or with task-irrelevant tactile stimuli (tactile training group), or were not adaptively trained, i.e., the active control group performed a 1-back task throughout all “training” sessions. The EEG power during the maintenance phase of the auditory WM task (Figure 1B) was compared between the low load (2-back task) and the high load condition (adaptive *n*-back task). We particularly focused on whether load-related changes in neuronal power differ between training groups, since we were interested in whether the neural correlates of WM processing at high load demands differ as a function of post-training performance efficiency. The increase in WM proficiency of trained participants was expected to result in WM processing changes. Importantly, if increased proficiency would result in mere activation differences in the networks activated during low load demands, no group differences would be expected during high load processing, as load levels were adjusted to the individual performance limit across groups. Instead, proficiency-related changes in WM processing should be present despite adjusted load levels across groups. Changes in WM networks were expected to be characterized by more efficient maintenance mechanisms. As suggested by previous studies, such increases in efficiency may be indicated for instance by a shift from attentional control processes to task-specific functions, involving perceptual processing, thus, by a shift from anterior to posterior activity (Buschkühl et al., 2012).

## Materials and Methods

### Participants

Forty-one healthy adults participated in the study and were pseudo-randomly assigned to three groups (cf. section “Experimental Procedure”). The data of two participants had to be discarded from the analyses, due to a decreased post-training performance relative to their pre-training performance (≥2SD from the mean pre-post-training difference). Thus, the final data included data sets of the remaining 39 participants (Table 1). Participants in the three groups did not differ regarding their sex (χ^2^(2) = 0.351, *p* = 0.839), age (*F*(2,36) = 0.484, *p* = 0.620), and education (more vs. less than ten years of schooling (χ^2^(2) = 2.60, *p* = 0.273). All participants were right-handed, had normal or corrected-to-normal vision, and normal hearing (self-report). None of the participants had a history of neurological or psychiatric disorders (self-report). Informed consent was obtained from all individual participants included in the study. Participants received monetary compensation for participation. The study was approved by the German Psychological Association.

**TABLE 1:** Demographic data of 39 participants^a^.

		Gender	Mean age in	Education (>10 years
	*n*	(females)	years (range)	of schooling)
AG	14	6	34 (23–55)	12
TG	11	6	30 (21–48)	9
CG	14	7	33 (21–55)	14

*^*a*^AG, auditory training group; TG, tactile training group; CG, active control group.*

Variables such as the perceived current stress level, wellbeing, and intelligence have been shown to affect WM performance (e.g., Ashby et al., 1999; Luethi et al., 2008; Luck and Vogel, 2013). To control for such confounding effects, all participants performed the German version of the Perceived Stress Questionnaire (PSQ-20: Levenstein et al., 1993; German modified version: Fliege et al., 2001) and a wellbeing scale [German: Habituelle Subjektive WohlBefindens Skala (HSWBS): Dalbert, 1992]. An estimation of the verbal intelligence score was obtained through the MWT-B (German Mehrfachwahl-Wortschatz-Test: Lehrl, 2005). The three groups did not differ in any of the assessed psychological variables (PSQ-R20: *F*(36) = 0.16, *p* = 0.854; HSWBS: *F*(35) = 0.03, *p* = 0.970; MWT-B: *F*(35) = 0.91, *p* = 0.412).

### Experimental Procedures

The reported data were part of a larger WM training study (Table 2), comprising pre-training EEG and MEG recordings, 4 sessions of behavioral WM training, post-training EEG and MEG recordings, and a final magnetic resonance imaging (MRI) session. Here, only behavioral and EEG data from the posttraining assessment and behavioral data from the training sessions will be reported. The other data have been published elsewhere. Previous publications investigated differences of the neural networks changed by WM training between sighted and congenital blind adults, analyzing the WM processing during a 2-back task prior to and after WM training (Gudi-Mindermann et al., 2018; Rimmele et al., 2019). In contrast, the present study focused on neurophysiological differences in WM processing at individual capacity limits, which have been enhanced (as shown on behavioral level; cf. section “Behavioral Performance”) by a previous training treatment, analyzing post-training WM processing of a demanding adaptive *n*-back task relative to a low load 2-back task.

**TABLE 2:** Full training procedure and study design^a^.

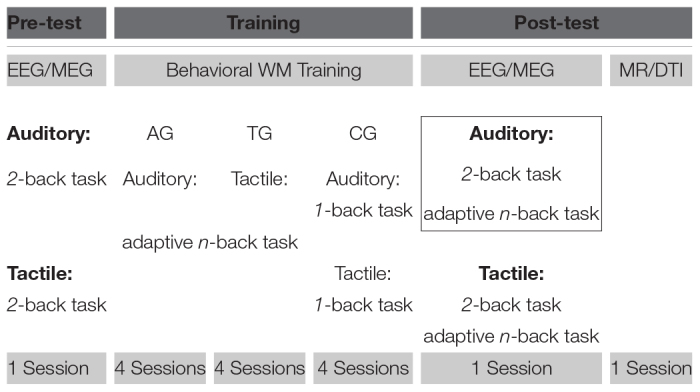

*^*a*^AG, auditory training group; TG, tactile training group; CG, active control group. Bold black box highlights the conditions that have been analyzed in the present paper. See text for details.*

In the pre-training sessions, participants performed an auditory and a tactile 2-back task. After each stimulus the participants had to indicate via button press with one of two fingers (index finger and middle finger of one hand; responding hand and finger were cross-balanced across participants) whether the stimulus was identical or not (target – 1/3: non-target – 2/3 distribution) to the stimulus presented 2 trials ago. Following the participants’ response, the next stimulus was presented after an inter-stimulus-interval (ISI) of a varying length, randomized between 1.3 and 1.7 s (Figure 1A).

Auditory stimuli consisted of the pseudo-word “befa” spoken by ten individuals (i.e., 10 different stimuli, 5 females and 5 males, stimulus duration: 450 ms, digitized at 44, 100 Hz and peak normalized at 65–75 dB). Participants had to match the speakers’ identity. The tactile stimuli were applied via Braille stimulators (QuaeroSys Medical Devices, Schotten, Germany). Five stimulators were attached to the fingers of one hand. The Braille stimulation generated a tactile motion percept by sequentially activating pairs of pins that were horizontally organized in four-by-two rows (4 × 112.5 ms, total stimulus duration 450 ms). The apparent motion either started at the fingertips (downward motion) or moved toward the fingertips (upward motion), resulting in ten different stimuli (i.e., 2 possible motion percepts at 5 fingers of one hand). Participants had to match the finger and the motion direction. Thus, the characteristics of the task, i.e., the length of each stimulus and the number of different stimuli, were held constant across the auditory and the tactile tasks.

Participants were pseudo-randomly assigned to three groups: auditory training group (AG), tactile training group (TG), and active control group (CG). In the AG and the TG an adaptive *n*-back task either with voices or with tactile stimuli was used during training. WM load was adaptively changed by adjusting the *n*-back level: The first block of the first training session started with a 2-back task. After every block the *n* was individually adjusted. The *n* increased by one if the performance exceeded both a hit rate of 70% and a correct rejection rate of 75%. The *n* decreased by one if the performance dropped below 60% in either hits or correct rejections. Otherwise, the *n* did not change. Spoken feedback after each block informed participants about their performance and announced the *n*-level of the upcoming block. Participants were instructed to strive toward the highest possible *n*-level and to prioritize accuracy over speed. Every consecutive training session started with the highest *n* that was reached at least three times during the previous training session. Each block consisted of 30 + *n* trials. For all *n*-back levels, sequences of targets and non-targets were constructed pseudo-randomly: The position of the targets (10 × *n*-back targets) varied randomly, while a fixed number of interfering distractors were incorporated (3 × “*n-minus-*1*”*-back lures and 3 × “*n-plus-*1”-back lures per block). A training session comprised 30 blocks and lasted typically for about 2 h, resulting in approximately 8 h of training per participant. All sessions took place on consecutive days or with no more than three days in between.

The AG was trained with auditory stimuli while the TG was trained with tactile stimuli. The CG performed a constant 1-back task throughout the four sessions, while the modality (auditory, tactile) of the task altered every session. An active control group was included to control for training-unrelated effects, such as familiarity with the stimulus material and procedure, as well as for non-specific effects resulting from being engaged in the training paradigm over several days. After training, the EEG was recorded during the auditory 2-back task (low load condition) and the auditory adaptive *n*-back task (high load condition). Note, with respect to the auditory task, the AG received a training in the same modality in which the post-training task was carried out, while the TG was trained in a modality that differed from the modality of the post-training task. In the following, to highlight this difference, the terms modality-specific training (AG) and modality-irrelevant training (TG) are used. The CG did not receive adaptive training.

In the high load condition, we aimed at testing participants’ performance at the individual’s capacity limit, irrespective of the group affiliation. Therefore, the *n*-level was set as follows: In case of the AG the adaptive *n*-back version started with the highest *n* that was reached at least three times during the last training session. In the case of TG the adaptive *n*-back version started 3 levels below the highest *n* that was reached at least three times during the last training session, to account for the modality switch between the tactile training and the auditory post-training task. For all CG participants the adaptive *n*-back version started with a 3-back task. The adaptive nature of the *n*-back task was kept for all three groups throughout the high load condition, to continuously adjust load demands to individual performance, while accounting for instantaneous learning and/or fatigue effects. The post-training EEG session comprised twelve blocks of the auditory 2-back task and fourteen blocks of the auditory adaptive *n*-back task. Participants were blindfolded during all EEG and training sessions, since this study was part of a bigger project, which additionally included blind individuals.

### EEG Data Acquisition and Preprocessing

The EEG was continuously recorded with 94 Ag/AgCl scalp electrodes (EasyCap GmbH, Herrsching, Germany), mounted in a cap according to the 10-5 system (Oostenveld and Praamstra, 2001). The electrooculogram (EOG) was recorded to monitor horizontal eye movements (potential difference between F9 and F10) and blinks (potential difference between Fp1 and an electrode placed below the left eye). The EEG signal was amplified with BrainAmp DC amplifiers (Brain Products GmbH, Gilching, Germany) and digitized using the BrainVision Recorder software (Brain Products GmbH). The analog EEG signal was sampled at 5,000 Hz, filtered on-line with a band pass of 0.1–1,000 Hz and then down-sampled on-line to 500 Hz. Impedances of all electrodes were kept below 10 kΩ.

The EEG data were preprocessed and analyzed using MATLAB 2016a (MathWorks) and the open source MATLAB toolbox Fieldtrip (Oostenveld et al., 2011)^1^. In all *n*-back tasks, the first *n* trials of a block were discarded from the analysis, since only the *n* + 1st stimulus can be compared to the stimulus presented *n* trials ago. Only trials with correct responses were considered. For preprocessing the continuous data were segmented ±1.9 s around stimulus onset. Data epochs for correct targets and non-targets were pooled together. Data were high pass filtered at 1 Hz. A standard automatic routine was applied to exclude data epochs contaminated by eye movements and muscle artifacts. The frequency ranges of the signal time courses that typically contain eye artifacts (1–15 Hz in 2 bipolar EOG channels) and muscle artifacts (110–140 Hz, in 94 data channels) were band-pass filtered and *z*-normalized per time point and electrode. The *z*-scores were averaged over electrodes, in order to accumulate evidence for artifacts, which typically occur in more than one electrode. Trials exceeding a predefined *z*-value (eye artifacts: *z* = 4; muscle artifacts: *z* = 15) were considered as artifacts and excluded. Line-noise was removed by subtracting 50-, 100-, and 150 Hz-Fourier components from the signal time course. Electrodes characterized by high variance across trials (visual inspection) were interpolated (spline interpolation; Perrin et al., 1989; mean number of removed electrodes: 2; range: 0–5). Last, all data channels were re-referenced to a common average reference.

Additionally, trials were excluded according to the following criteria: First, all blocks in which the *n*-back level dropped to *n* = 2 (low load condition) were excluded from the high load condition to avoid identical *n*-back levels between load conditions. Second, the adaptively changing *n*-back levels allowed to instantaneously (block-wise) adjust the load demands (*n*-back level) to individual capacity limits throughout the high load condition. To avoid *n*-back levels outside individual capacity limits, which might have occurred during the adjusting process, blocks exceeding individual capacity limits (accuracy rates < 60%) and falling below individual capacity limits (accuracy rates > 90%) were excluded. The average number of included trials per participant was 182 trials (47%) [SD = 42 (11%)] in the 2-back low load condition and 103 trials (25%) [SD = 45 (11%)] in the *n*-back high load condition.

### Data Analyses

#### Estimation of Spectral Power and Source Reconstruction

Discrete Fourier Transforms of EEG maintenance activity (−1.1 to −0.2 s; Figure 1B) were calculated at 2.5–100 Hz (segment length: 0.4 s; segment shift: 0.08 s; frequency resolution: 2.5 Hz). The cross-spectrum of the Fourier transformed time segments was retrieved per participant for each electrode and frequency bin in both conditions (low and high load) (Genovese et al., 2002; Nolte et al., 2004). Cross-spectra were averaged across time segments and across trials.

The standard Montreal Neurological Institute (MNI) average brain was used for source reconstruction. Based on the cortical surface of the standard head model a grid was created as a set of 3,000 as equally as possible distributed source points. Every source point represented an equivalent current dipole (cf. Cromer et al., 2010). A standardized three-dimensional map of electrode locations was generated. First, the locations of the 94 employed electrodes were measured three times on a template plastic head, using the ultrasonic Elpos system (zebris Medical GmbH, Isny im Allgäu, Germany). Next, to minimize measurement errors, the positions of the three independent measurements were averaged and centered along the midline. These standard electrode locations and the standard grid model were used to compute a standard leadfield matrix. If needed, the standard leadfield was individually adjusted by excluding noisy channels, characterized by high variance across trials, which were interpolated during the individual preprocessing procedure (cf. section “EEG Data Acquisition and Preprocessing”). Exact Low-Resolution Brain Electromagnetic Tomography (eLoreta), a discrete, linear, three-dimensional distributed, weighted minimum norm inverse solution, was used to calculate a spatial filter based on the individually adjusted leadfields. The eLoreta spatial filter localizes the power distribution of the EEG signal with exact maxima for single dipoles (Shafi et al., 2007). The real parts of the cross-spectrum were projected into source space by multiplication with the spatial filter for every source point. Source space power was defined as the maximal eigenvalue of the cross-spectrum, over the three dipole directions. The resulting value per source point represented the power value at that source point (cf. Polomac et al., 2015).

**FIGURE 2:**
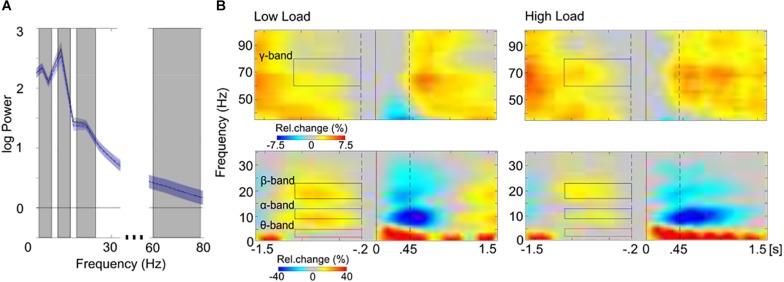
Spectral **(A)** and spectro-temporal **(B)** distribution of maintenance activity. **(A)** The mean overall power spectrum (log power; SEM, shaded area) is displayed separately for the low load (black line) and the high load (blue dashed line) conditions. Power values of the averaged maintenance activity (–1.1 to –0.2 s) were averaged across all voxels and across participants of all training groups. The gray boxes highlight the pre-selected frequency-bands that were used for further analyses (from left to right: theta, 2.5–5 Hz; alpha, 10–12.5 Hz; beta 17.5–22.5 Hz; gamma 60–80 Hz; the spectral resolution of 2.5 Hz is considered). Note, oscillatory peaks are present in all pre-selected frequency bands in the spectral profile of the averaged maintenance activity. **(B)** The sensor-space time-frequency representations of low (left; <36 Hz) and high (right; >36 Hz) frequencies are depicted separately for the low load (top) and the high load (bottom) conditions, averaged across all electrodes and across participants of all training groups. Stimulus-locked (0 s, solid black line) spectral power from –1.6 to 1.6 s (stimulus duration 0.45 s, right dashed black line) is expressed in percent change (Rel. change in%) relative to baseline activity (–0.2, left dashed black line; to 0 s, solid black line). Colored boxes highlight the pre-selected frequency-bands that were used for further analyses (left: red, theta; blue, alpha; black, beta; right: blue, gamma). Note the sustained increase of band-specific activity during the maintenance period (–1.1 to –0.2 s, colored boxes). In further analyses only this maintenance period was analyzed, since the rest of the trial may be overlaid by reaction times (cf. Figure 1B).

Finally, source power estimates were log-transformed and averaged over frequency ranges of interest, resulting in four frequency bands that were used for further analyses: theta (2.5–5 Hz), alpha (10–12.5 Hz), beta (17.5–22.5 Hz), and gamma (60–80 Hz). These frequency ranges were selected to best represent the core of the theta-, alpha-, beta-, and gamma-bands (cf. Kubicki et al., 1979), given the frequency resolution of 2.5 Hz. The inspection of the overall power spectrum (averaged across voxels) showed that the pre-selected frequency bands in fact captured oscillatory peaks in the spectral profile of the averaged maintenance activity (Figure 2A). Furthermore, for additional confirmation that oscillatory activity in the pre-selected frequency bands represented sustained WM-relevant activity, time-frequency representations (TFRs) of maintenance activity were calculated (overall power across sensors was analyzed in sensor space). The spectral parameters were estimated using multitapers for a trial length of −1.6 to 1.6 s around stimulus onset (Figure 1B) with a spectral smoothing of 4.5 Hz for frequencies below 36 Hz and with a spectral smoothing of 8 Hz for frequencies above 36 Hz. For TFRs a sliding time window of 0.4 s was used that was stepped by 50 ms through the trials. TFRs were normalized by 200 ms pre-stimulus activity (baseline). Figure 2B shows TFRs of the WM maintenance as percent change relative to baseline. Note, the pre-stimulus baseline, which itself is part of the maintenance period, most probably has reduced WM-relevant activity during the maintenance period. Despite this constraint the inspection of the temporal domain of maintenance activity confirmed that the pre-selected frequency bands represented frequency ranges of the sustained, narrow-band maintenance activity (Figure 2B).

#### Statistical Analyses

Analyses of behavioral data were conducted using R (R Core Team, 2016) implemented in RStudio (v0.99.486; R Studio Team, 2015)^2^. Behavioral training effects were tested by analyzing the mean WM capacity, represented by the individual *n*-back levels. A one-way analysis of variance (ANOVA) was run on the mean *n*-back levels that were reached during the adaptive high load condition.

**FIGURE 3:**
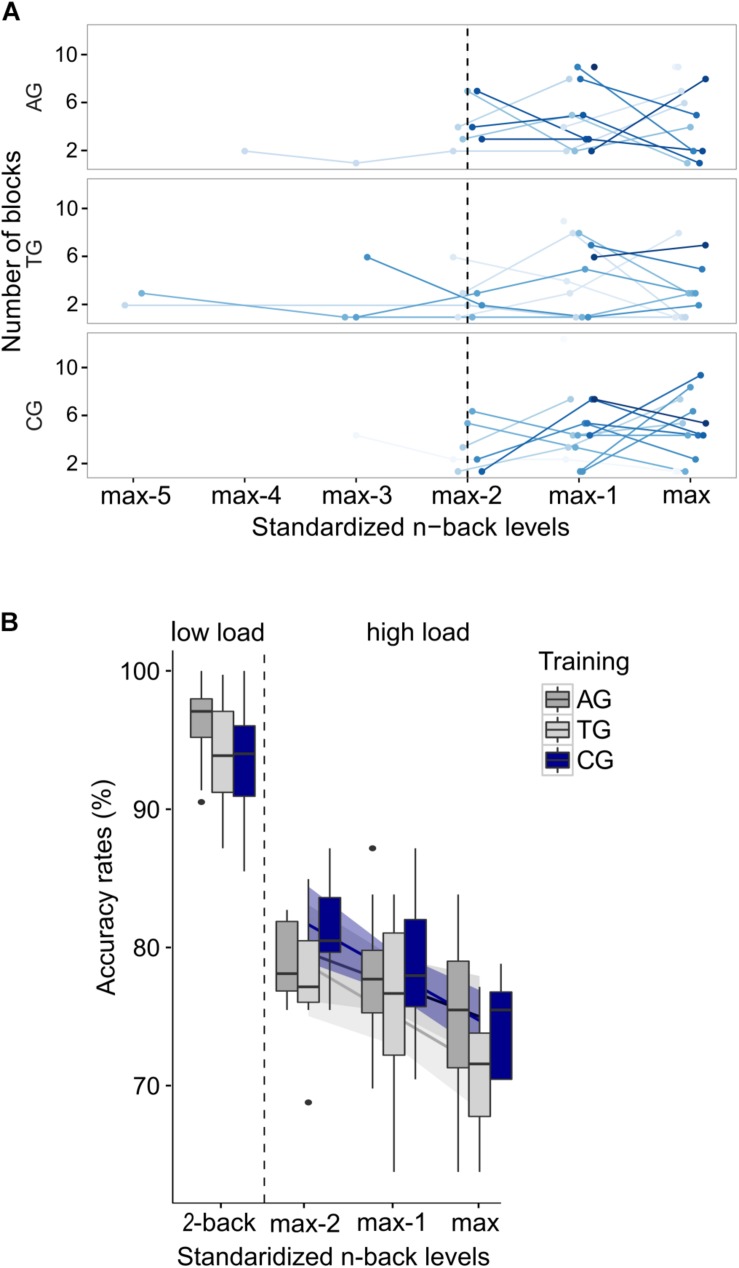
Preprocessing in behavioral *n*-back data. **(A)** Individual frequency distribution of all prevailing recoded *n*-back levels is depicted for each group (“max” represents the highest *n*-back level, an individual reached in the adaptive high load condition; “max–1,” “max–2,” etc. represent the remaining *n*-back levels, coded in a descending order). Single lines (blue color-coding) represent individual data. Data from the three individually highest *n*-back levels (left of the dotted line) were included in the following analyses. **(B)** Mean accuracy rates are shown separately for each group and load condition; and for each *n*-back level in the adaptive high load condition. Error bars indicate one standard error of the corresponding mean.

Prior to comparing accuracy rates between load conditions, the load demands in the *adaptive* high load condition had to be adjusted to represent individual capacity limits, despite differing *n*-back levels between participants (cf. section “Behavioral Results”), differing training experience between participants, and differing WM capacity between participants. To this end, three preprocessing steps were applied. The first two steps were identical to those used for preprocessing EEG data. First, all blocks in which the *n*-back level dropped to *n* = 2 (low load condition) were excluded from the high load condition to avoid identical *n*-back levels between load conditions. Second, the adaptively changing *n*-back levels allowed to instantaneously (block-wise) adjust the load demands (*n*-back level) to individual capacity limits throughout the high load condition. To avoid *n*-back levels outside individual capacity limits, which might have occurred during the adjusting process, blocks exceeding individual capacity limits (accuracy rates <60%) and falling below individual capacity limits (accuracy rates >90%) were excluded. The average number of included blocks per participant was 11 blocks (330 trials) out of 14 blocks (420 trials) in total. Finally, performance is by definition negatively correlated with the load factor *n*. Thus, in a third preprocessing step, the variance in accuracy, introduced by various *n*-back levels within the adaptive high load condition, had to be accounted for. To this end, the individual *n*-back levels were recoded for standardization: Each individual’s highest *n*-back level during the adaptive high load condition represented her/his individual limit in WM capacity. Starting from this personal maximum, the remaining *n*-back levels were coded in a descending order (max, max–1, max–2, etc.). The amount of *n*-back levels processed during the high load condition varied between 1 and 6 levels (Figure 3A). However, the highest number of different *n*-back levels processed by more than one participant in all three groups was 3, i.e.: max, max–1, and max–2 (Figure 3A). All blocks outside these *n*-back levels (i.e., max–3, max–4, max–5) were excluded from further analyses^3^.

Having adjusted the load demands within the high load condition a mixed logistic regression model (generalized linear mixed model, GLMM with a logit link function) was run on high load accuracy rates to ensure that the adjusting procedure worked and, thus, similar requirements prevailed within the high load condition across groups and participants despite differing *n*-back levels. To this end, the covariate *recoded n-back-levels* (max, max-1, max-2) and the predicting variable *group* (AG, TG, CG) were included as fixed effects. The covariate *recoded n-back-levels* was, furthermore, included into the random effects structure, allowing for random intercepts and slopes for each subject. Significance of fixed effects was tested with Wald χ^2^ tests. The GLMM confirmed that following the adjusting procedure and after having accounted for the covariate (χ^2^(1) = 16.01, *p* < 0.001) performance within the high load condition no longer differed between groups (χ^2^(2) = 4.45, *p* = 0.108; Figure 3B), indicating comparable load demands between groups irrespective of differing *n*-back levels. Note that prior to the adjusting procedure performance between groups did differ significantly within the high load condition (χ^2^(2) = 6.16, *p* = 0.046; not shown) despite accounting for the covariate (χ^2^(1) = 10.16, *p* = 0.001). This indicates the natural differences in load demands due to differing *n*-back levels reached in different groups during the adaptive procedure of the task.

The key interest in the present study was to investigate whether WM training alters neural mechanisms when load demands approach individual capacity limits. Particularly, we expected neural mechanisms under high load demands to be altered in response to different types of WM training. Thus, source power of WM maintenance activity was contrasted between the low and the high load condition: Pow_diff_ = Pow_high–load_ – Pow_low–load_, resulting in load-related power changes. Importantly, as detailed above, in the adaptive high load condition, load demands had been adjusted prior to the analysis in order to represent individual capacity limits, eliminating the confound of interindividual differences in WM capacity across trained and non-trained participants.

To statistically evaluate differences in WM load effects in the auditory WM task between groups (AG, TG, CG), for each of the four frequency bands, cluster-based permutation tests on load-related power changes were employed (1,000 iterations; Maris and Oostenveld, 2007), with group affiliation being randomly permuted across participants. Specifically, two planned group contrasts were performed: (1) the auditory training group was contrasted with both other groups (AG_Powdiff_ vs. TG_Powdiff_ & CG_Powdiff_), to analyze the impact of modality-specific WM training on the load effect; (2) both, i.e., auditory and tactile, training groups were contrasted with the active control group (AG_Powdiff_ & TG_Powdiff_ vs. CG_Powdiff_) to analyze the general impact of WM training on the load effect, irrespective of training modality. Spatial clusters were formed of adjacent source points with *t*-values below *p* < 0.05. The *t*-values within clusters were summed. Clusters, which exceeded 95% of the largest summed *t*-values from the permutation distribution, were considered statistically significant when *p* < 0.025 (two-sided *t*-tests).

## Results

### Behavioral Performance

**FIGURE 4:**
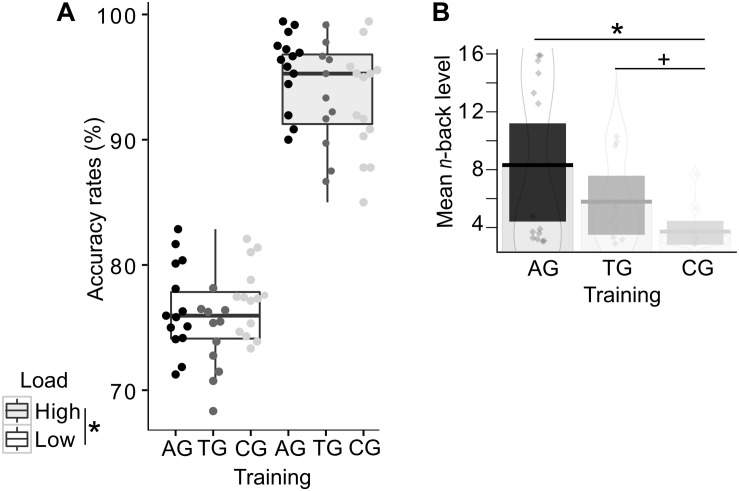
Behavioral training effects in the auditory *n*-back task. Single dots represent individual data. **(A)** Accuracy rates are displayed separately for each group and load condition, demonstrating the load effect irrespective of group affiliation. Color-coded dots represent individual data. Error bars indicate the standard error of the corresponding mean. **(B)** The mean absolute *n*-back level (i.e., not recoded) is shown per group, with highest mean *n*-back levels in the auditory training group (AG), followed by the tactile training group (TG), and finally, the active control group (CG). Both training groups reached higher *n*-back levels (AG: significance; TG: tendency) compared to the active control group (CG). Asterisks indicate significance, the plus-symbol indicates marginal significance.

In order to test the effects of load on WM performance, accuracy rates were compared with a GLMM, including the predicting variables *WM load* (low and high load) and *group* (AG, TG, CG) as fixed effects. The *WM load* was additionally included into the random effects structure, allowing for random intercepts and slopes for each subject. The GLMM confirmed the expected main effect of load (χ^2^(1) = 60.67, *p* < 0.001; Figure 4A), reflecting the expected drop in performance in the high load condition across all groups. The predicting variables *group* (χ^2^(2) = 4.29, *p* = 0.117) and the interaction between *WM load* and *group* (χ^2^(2) = 4.36, *p* = 0.113) were not significant. Note that the absent interaction effect between *WM load* and *group* provides no conclusions about training-related performance changes, as the *n*-back levels were adjusted. Instead, in this analysis the absent interaction effect again confirms that the adjusting procedure (cf. section “Data Analyses”) was successful and load demands, as indicated by accuracy, were comparable across groups despite differing *n*-back levels in the high load condition.

In order to address training-related performance differences, we analyzed the mean WM capacity, represented by the mean *n*-back levels reached during the adaptive high load condition. A one-way ANOVA revealed a significant effect of group (*F*(2,36) = 6.21, *p* = 0.005; η^2^_*p*_ = 0.204; Figure 4B), reflecting a training-related increase in WM capacity. The consecutive Bonferroni-corrected *post hoc* two-sided *t*-tests showed the expected performance advantages following modality-specific WM training, as indicated by higher *n*-back levels in the auditory training group relative to the control group (AG vs. CG: *t*(26) = 3.23, *p* = 0.010). The tactile training resulted in marginally significant higher *n*-back levels relative to the control group (TG vs. CG: *t*(23) = 2.56, *p* = 0.053). AG and TG did not significantly differ in their mean *n*-back levels (AG vs. TG: *t*(23) = 1.58, *p* = 0.386).

**FIGURE 5:**
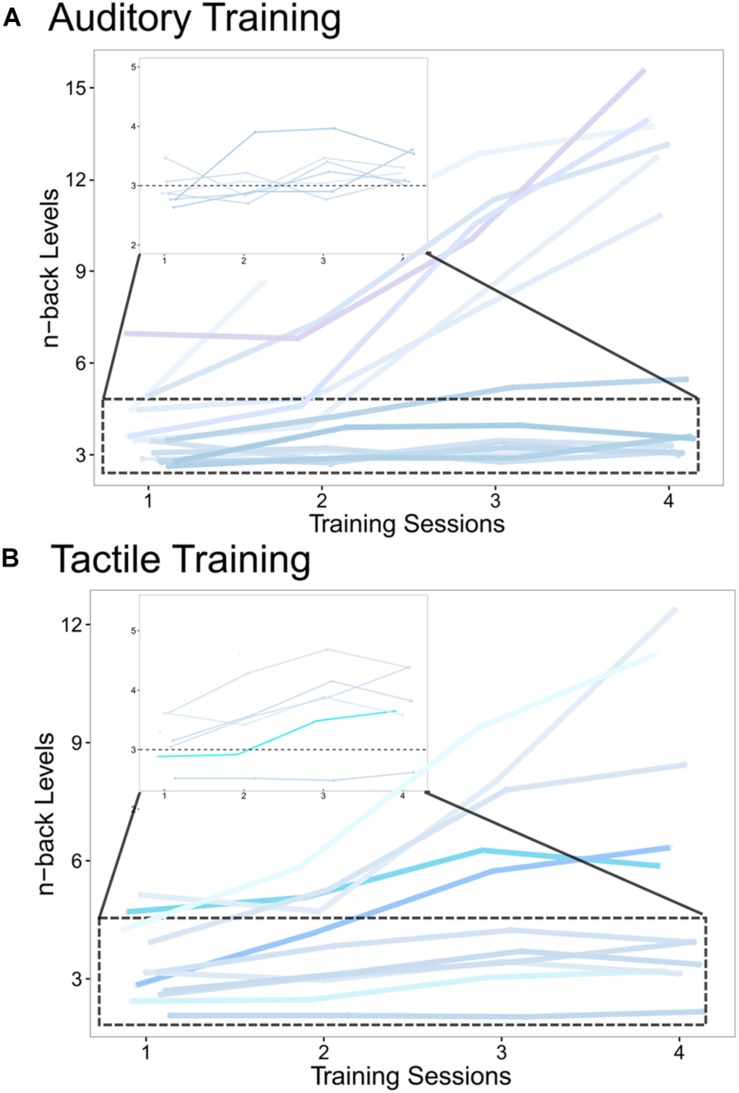
Individual training tracks measured during the 4 training sessions of **(A)** the auditory training group and **(B)** the tactile training group. The individual courses (blue color-coding) of *n*-back levels, averaged over 30 blocks per training session, are shown for all four training sessions, demonstrating the growing WM capacity with training progress. The zoomed in area (dotted box) shows participants with more moderate *n*-back increases. The horizontal dotted line at 3-back level serves as a guide to highlight capacity increases.

Additionally, to ensure that no baseline differences existed between groups before training, a one-way ANOVA was run on pre-training 2-back accuracy rates, revealing no significant effect of group (*F*(2,36) = 2.03, *p* = 0.147; η^2^_*p*_ = 0.101; data not shown). The individual training tracks (courses of *n*-back levels over time across the four training sessions) of AG and TG are shown in Figure 5. No training tracks can be shown for CG, since this group performed only the 1-back task with no changes in *n*-back levels throughout the four sessions.

### Post-training Load-Related Source Power Changes

**FIGURE 6:**
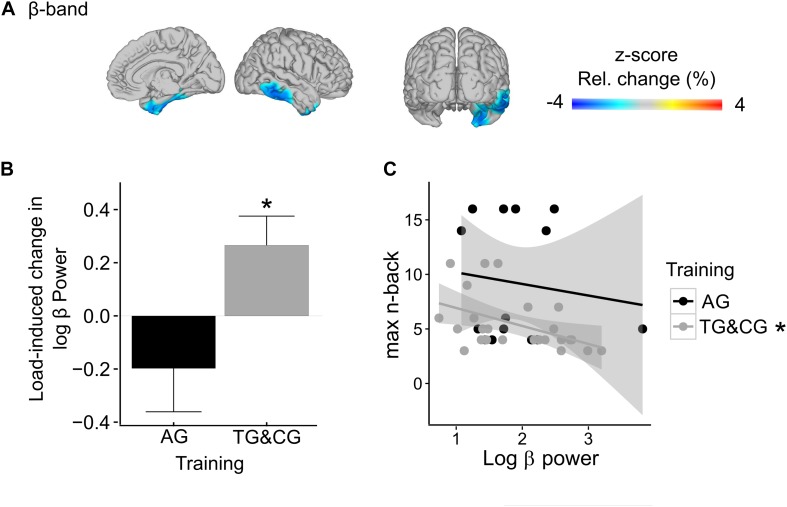
Training-related differences in WM load effects (AG vs. TG&CG)* in beta-band power (17.5–22.5 Hz). **(A)** The observed significant interaction between training and WM load in the right medial temporal lobe is projected onto a glass brain. In interaction-sensitive voxels, color-coding represents *z*-scores in power change between conditions. Note, light-blue to dark-blue colors indicate reduced load effects in the AG relative to the TG and CG. **(B)** The bar graph shows the load-related power changes separately for AG and TG and CG, pooled across all interaction-sensitive voxels. **(C)** The relationship between beta-band power in the right medial temporal lobe and maximally reached *n*-back levels in the adaptive high-load condition is depicted separately for AG and TG and CG, showing a negative correlation for the tactile training group and the control group, but not for the auditory training group. Asterisks indicate significance. * AG, auditory WM training group; TG, tactile WM training group; CG, active control group.

We observed a statistical trend for reduced load effects in beta-band power in sensory processing areas in participants with modality-specific WM training relative to both other groups (AG vs. TG&CG; marginally significant cluster, *p* = 0.069; Figure 6A). The identified cluster included the right medial temporal lobe, extending along the superior temporal sulcus. Group-specific *post hoc* analyses (Bonferroni-corrected) confirmed that beta-band power in the identified cluster, pooled across all significant voxels, was significantly increased in the high load condition compared to the low load condition in both the TG and CG (*t*(24) = 3.33, *p* = 0.003; Figure 6B; separately for TG and CG: TG: *t*(10) = 2.21, *p* = 0.052; CG: *t*(13) = 2.55, *p* = 0.024), but not in the AG (*t*(13) = −1.58, *p* = 0.137; Figure 6B). Furthermore, in the high load condition, beta-band power in the identified cluster was negatively correlated with the maximally reached absolute *n*-back level (Bonferroni-corrected) in the TG and CG (*r* = −0.47, *p* = 0.019; Figure 6C), but not in the AG (*r* = −0.13, *p* = 0.652; Figure 6C). No further differences in load-related power changes were observed between the AG vs. TG and CG, neither in the theta- (in all identified clusters all *p* > 0.363), nor in the alpha- (in all identified clusters all *p* > 0.462), nor in the gamma-band (in all identified clusters all *p* > 0.155). Finally, no general impact of WM training on the load effect, irrespective of the modality, was observed when contrasting both training groups with the control group (AG&TG vs. CG; in all identified clusters all *p* > 0.197).

### Load-Related Modulations in Source Power

**FIGURE 7:**
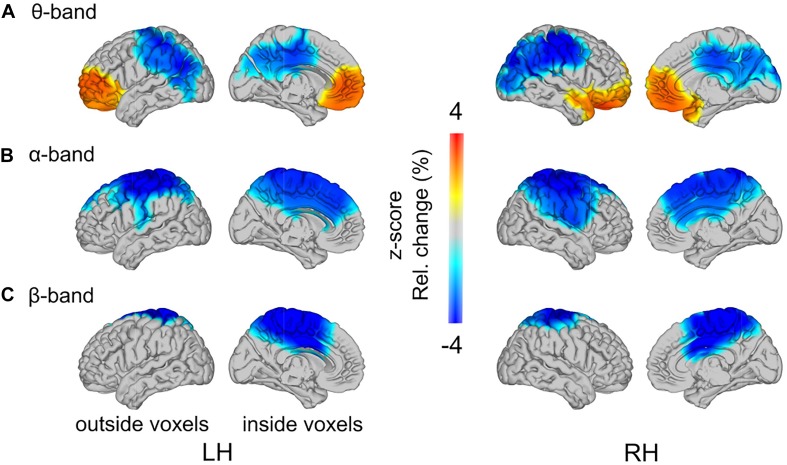
Load-related power changes in **(A)** theta- (2.5–5 Hz), **(B)** alpha- (10–12.5 Hz), and **(C)** beta-band (17.5–22.5 Hz), across all training groups, are projected onto a glass brain. Outside and inside voxels are depicted for the left (LH) and right (RH) hemispheres. Color-coding represents *z*-scores in power change between low load and high load conditions.

**FIGURE 8:**
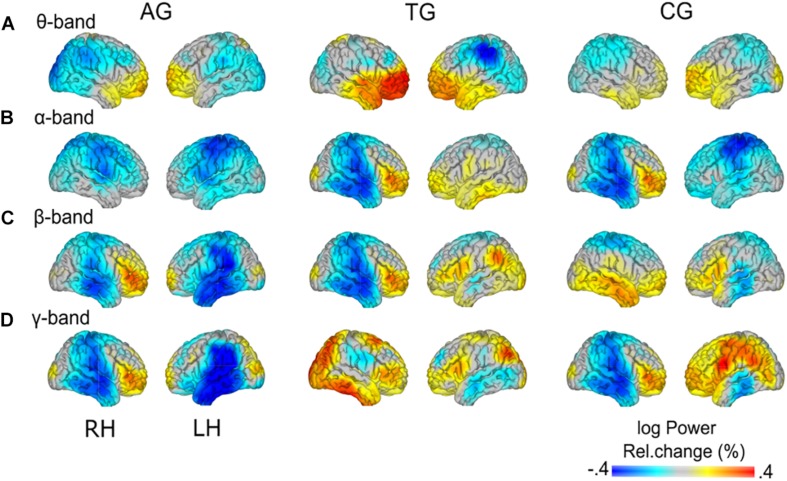
Group-specific load effects. Load-related power changes (log power) are depicted for **(A)** theta- (2.5–5 Hz), **(B)** alpha- (10–12.5 Hz), **(C)** beta- (17.5–22.5 Hz), and **(D)** gamma-band (60–80 Hz), separately for the three training groups (AG, auditory training group; TG, tactile training group; CG, active control group). Note the group-specific differences in load effects (cf. Figure 7) vs. similarities (cf. Figure 8) in load effects across groups.

As reported above, load-related power changes were modulated by WM training only at trend-level. Thus, the main effect of WM load was additionally analyzed across all participants, irrespective of group affiliation. In the theta-band, one cluster with positive *t*-values (*p* = 0.015) and one cluster with negative *t*-values (*p* = 0.002) reached significance (Figure 7A). In the high load relative to the low load condition, theta-band power was increased at bilateral frontal poles, spreading to left dorsal and right ventral regions in the frontal lobe. The load-related theta power decrease was localized to the parietal cortex, with strongest desynchronization over bilateral inferior parietal lobules. In the alpha-band power, one significant cluster with negative *t*-values (*p* = 0.002) was found (Figure 7B). The load-related alpha-band power was decreased over bilateral centro-parietal regions broadly spreading around pre- and postcentral gyri. In the beta-band power, a significant cluster with negative *t*-values (*p* = 0.010) was observed over bilateral central parts of the cingulate gyri, including bilaterally the precuneus (Figure 7C). Finally, in the gamma-band power no significant clusters were observed (in all identified clusters all *p* > 0.284). For full transparency, furthermore, the low-high load contrast is displayed separately for each training group (Figure 8).

## Discussion

The present study investigated the effects of WM training by comparing neural mechanisms of auditory WM performance in different training groups under high load conditions. The behavioral results confirmed that the WM training was successful compared to the active control group, showing performance increases that suggest an extended WM capacity particularly in the modality-specific (auditory) WM training group. The EEG results revealed two main findings. First, participants who received modality-irrelevant tactile WM training and those who did not receive adaptive WM training (control group), showed load-related increases in beta-band power in the right temporal lobe, when performing the auditory WM task. These changes were not observed in participants who were trained with task-relevant auditory stimuli. This provides evidence that for individuals with higher WM proficiency neural processing was modulated, possibly underlying increases in individual WM capacity. However, note that the main effect of this analysis was only observed at trend level and requires follow-up research for confirmation. Second, we found load-related power changes across all groups in the theta-, alpha-, and beta-band: With increasing load demands, theta-band power increased at bilateral frontal poles, while power decreased in posterior theta-, centro-parietal alpha-, and mid-central beta-band. This suggests that WM processing at high load demands requires both a strengthening of task-relevant processing as well as an attenuation of task-irrelevant processing.

### Behavioral Training Effects

As expected, relative to the control group, the increases in auditory WM capacity in the training group, as indicated by the mean *n*-back levels reached during the adaptive post-training task, was significant following modality-specific auditory training, and marginally significant following modality-irrelevant tactile training. We, thus, observed a marginal transfer of tactile training on auditory WM. The observed advantage of modality-specificity (i.e., a significant vs. a marginally significant WM capacity increase relative to a control group) between training and task is consistent with previous findings. For example, Schneiders et al. (2011) observed a greater training gain in a visual WM task following visual training as compared to auditory training. Typically, WM training effects have been described as narrow, declining with transfer demands (for reviews see Melby-Lervag and Hulme, 2013, 2016; Soveri et al., 2017; but see Au et al., 2015, 2016). Nevertheless, our findings indicate that both modality-specific auditory and modality-irrelevant tactile training paradigms improved WM processing, the latter, however, at a marginally significant level.

### Training-Related Differences in WM Load Effects

Our main focus in the present study was on modulations of WM load effects, comparing groups with different post-training WM performance. Individuals who received WM training with task-irrelevant tactile stimuli (TG) and non-adaptive training (CG), relative to those who received WM training with task-specific auditory stimuli (AG), showed load-induced beta-band increases in the right medial temporal lobe, extending along the superior temporal sulcus. This increase in beta-band power, furthermore, correlated negatively with the absolute *n*-back levels that were maximally reached under the high load condition.

These results suggest that participants who did not receive modality-specific WM training (TG and CG) required additional activation of right medial temporal regions as load demands increased. The medial temporal lobe (including hippocampal structures together with anatomically adjacent cortical regions) has been reliably associated with both encoding and retrieval of information (for a review see Simons and Spiers, 2003), being particularly critical for rapid learning of new episodic information (Patterson et al., 2007). Furthermore, the observed activation in the right medial temporal lobe extended along the superior temporal sulcus, an area known to be voice-selective, with an emphasis on the right hemisphere (Belin et al., 2000, 2002). The task-relevant features used in the auditory verbal *n*-back task of the present study were voices, which participants had to match regarding the speakers’ identities. Thus, the increased activation in these memory- and voice-relevant areas seems to reflect additional support of WM processing under high load demands. These considerations are in line with a study by Leiberg et al. (2006). In an auditory Sternberg task with syllables spoken by a natural voice, the authors found a load-induced increase in beta-band power over right temporal MEG sensors during the maintenance period. Leiberg et al. (2006) related the load-induced parametric beta-band enhancements to the maintenance of an increased stimulus set, suggesting that beta-band power codes for the representations of task-relevant stimulus features. Given that we adjusted the task-difficulty to a comparable level across individuals and groups (cf. section “Data Analyses”), these results confirm our hypothesis. They show that, despite all individuals performing at their capacity limits (adjusted load-levels), participants of the tactile training group and the control group recruited additional brain areas for successful WM processing at high load demands compared to participants with modality-specific training. Effects might result from more efficient maintenance of task-relevant stimulus representations, following modality-specific auditory training. Probably a higher experience with task-relevant auditory stimuli facilitated perceptual processing, supporting maintenance processes. The fact that the beta-band activity may be related to processing efficiency is further reflected in the negative correlation between the absolute *n*-back level and beta-band power in the right temporal lobe in participants of the tactile training group and the active control group. That is, participants with highest gains in performance, as indicated by higher *n*-back levels reached during the adaptive high load condition, showed the least load-induced beta-band increases, and thus, the least need for additional activation in the right medial temporal lobe. Possibly, a training-induced efficiency in maintenance processes results in a more efficient resource allocation from perceptual to WM-related processing.

These group-specific differences in beta-band power were right-lateralized, thus being in accordance with literature on voice-selective regions (Belin et al., 2000, 2002), and more general, in accordance with literature on hemispheric lateralization of the auditory cortex. There is a higher selectivity of the right auditory cortex for the processing of slow spectral aspects of auditory input, such as speech prosody or pitch variations, over fast temporal aspects, along with a complementary specialization of the left auditory cortex (for review: Poeppel, 2001; Zatorre et al., 2002; Assaneo et al., 2019). The right lateralization of the observed effect corresponds to this functional differentiation, as in our study voices, i.e., sounds’ spectral resolution, had to be maintained and discriminated.

This finding, however, has been observed only at statistical trend level, and has to be interpreted with caution. Nevertheless, given this marginally significant result finds support in further research, these data would provide neurophysiological evidence for the previously reported “narrowness” of behavioral training effects (Melby-Lervag and Hulme, 2013, 2016). Narrow training effects are indicated by attenuated training gains with transfer to other sensory modalities (Schneiders et al., 2011) or similar tasks (Dahlin et al., 2008). Our results demonstrate that the narrowness of training effects may be related to a training-induced increase in efficiency to maintain representations of task-relevant features, particularly following modality-specific WM training. Importantly, as reflected in group-specific WM load effects during the WM *maintenance period*, the here reported training-related benefits go beyond previously reported mere encoding advantages (e.g., Lustig and Flegal, 2008).

### WM Load Effects

Working memory load effects across all participants and groups were observed in theta-, alpha-, and beta-band power, but not in gamma-band power.

In line with previous studies (Krause et al., 2000; Jensen and Tesche, 2002; Deiber et al., 2007; Notebaert and Verguts, 2008), we found that theta-band oscillatory activity increased with WM load over bilateral prefrontal regions. The load-related increases in frontal theta-band power have been functionally related to enhanced requirements of cognitive control and executive functioning at higher task demands (Sauseng et al., 2010; Hellbernd and Sammler, 2016). The distribution of voxels with significant effects, comprising the frontal theta-band increase, spread from bilateral mid frontal poles in the left hemisphere to *dorsal* regions, and in the right hemisphere to *ventral* regions. Neuroimaging studies, aimed at classifying activation patterns among WM-related regions, have linked dorsal frontal activation to executive processes, necessary for a continuous updating and maintenance of the sequential order of items in WM (D’Esposito et al., 1998; Owen, 2000; Lisman, 2010; Heusser et al., 2016). Right-lateralized ventral frontal activation was associated with the manipulation of the stored information (Wager and Smith, 2003), particularly including selection and evaluation of WM items (Owen et al., 2005). It is reasonable to assume that under high load conditions both types of these executive functions – continuous updating and maintenance as well as manipulation – are required to a larger extent during WM performance. Thus, the observed asymmetric spreading of theta-band increases toward left dorsal and right ventral regions possibly reflects distinct sub-functions involved in WM processing.

Additionally, we observed load-induced theta-band decreases in the parietal cortex. Theta-band decreases in regions other than frontal cortex have been previously reported (Howard et al., 2003; Meltzer et al., 2008; van Vugt et al., 2010; but see, Raghavachari et al., 2006; Sauseng et al., 2010). The strongest theta-band desynchronizations were observed over bilateral inferior parietal lobules. Bilateral supramarginal gyri, located in the inferior parietal lobules, have been shown to contribute to phonological decisions (McDermott et al., 2003; Shalom and Poeppel, 2008; Hartwigsen et al., 2010). The stimuli in the auditory *n*-back task implemented in the present study consisted of a pseudo-word spoken by different individuals. That is, the speakers’ voices, thus, the sounds (i.e., phonology) of pseudo-words had to be maintained and discriminated. Hence, theta-band decreases over bilateral inferior parietal lobules are probably related to more efficient processing of voice stimuli. These considerations are in line with an fMRI study by Schon et al. (2008), who observed gradual load-induced BOLD-signal decreases in left lateral temporal regions along with prefrontal activation increases in a dual *n*-back task, in which visuospatial and auditory information had to be maintained simultaneously. The authors related this anterior-posterior shift in BOLD activity to a load-related shift from perceptually dominated processing to memory processing. Thus, similarly as Jaeggi et al. (2007), we speculate that the observed load-induced desynchronizations over inferior parietal regions may indicate a shift from phonological processing to enhanced cognitive control, as indicted by load-induced frontal theta-band increases.

In the alpha-band, oscillatory power decreased with WM load over bilateral centro-parietal regions. These load-induced alpha-band desynchronizations are in good agreement with previous studies implementing the *n*-back paradigm (Gevins et al., 1997; Deiber et al., 2007; Schuster et al., 2018). Together with previous evidence that parietal regions, in addition to frontal regions, constitute the core WM network (for a review see Eriksson et al., 2015), our data suggest that the observed desynchronizations in bilateral centro-parietal alpha-band power reflect overall enhanced engagement with high processing demands (cf. Klimesch et al., 2007).

Interestingly, while our data support the interpretation of enhanced involvement of both frontal and centro-parietal regions with increasing load demands, the underlying neural correlates differ in their spectral profiles, as indicated by load-induced frontal *theta-band increases* versus load-induced centro-parietal *alpha-band decreases*. However, the relatively low frequency resolution of 2.5 Hz applied in our analyses (cf. section “Data Analyses”) may constrain our findings particularly in the low frequency range.

In the beta-band, we found WM load-induced power decreases over bilateral mid-central regions. Although in contrast to some studies, reporting load-related increases of WM-relevant oscillatory activity in *n*-back tasks (Deiber et al., 2007; Larrouy-Maestri et al., 2013), our findings are consistent with the frequent reports of load-related beta-band desynchronizations, mainly at medial regions (along the anterior-posterior-axis) including the cingulate cortex (Pesonen et al., 2007; Brookes et al., 2011; Palomaki et al., 2012; Takei et al., 2016; Scharinger et al., 2017). Interestingly, Brookes et al. (2011) stressed the largely overlapping spatial distribution of load-induced beta-band desynchronizations in WM processing with regions comprising the default mode network (DMN). The DMN commonly involves the medial prefrontal cortex, bilateral inferior parietal lobules, and, particularly, medially located posterior and anterior cingulate cortices (Shulman et al., 1997; Raichle et al., 2001). This anatomically confined brain system has been characterized by spontaneous activity during cognitive disengagement from the external world, and complementary, by task-induced deactivations (Gusnard and Raichle, 2001; Buckner et al., 2008). Of particular relevance for the present results is that simultaneous EEG and fMRI recordings have linked the DMN, typically studied with neuroimaging methods, mainly to *beta-band* oscillatory activity (Laufs et al., 2003; Mantini et al., 2007; Brookes et al., 2011). Accordingly, we assume that the observed load-induced decreases in beta-band activity over medial centro-parietal regions might reflect a task-related attenuation of default mode functioning. Similarly, in fMRI studies, activity in the DMN has been observed to decrease with increasing WM load (Esposito et al., 2006; Woodward et al., 2006; Park et al., 2011; Piccoli et al., 2015).

Apparently, when it comes to highly demanding cognitive tasks, complementary mechanisms in terms of suppression of task-irrelevant processing and strengthening of task-related processing seem to be relevant for successful performance. As for alpha-band synchronization (Klimesch et al., 2007), we hypothesize that beta-band desynchronizations are involved in inhibitory control. More specifically, alpha-band synchronizations have been linked to *exogenously* driven top-down inhibition to prevent interference from task-irrelevant sensory input or task-irrelevant sensory/external processing (Klimesch et al., 2007). For beta-band oscillations, we speculate that desynchronizations over regions defining the DMN reflect an *endogenously* driven top-down inhibitory system to prevent interference from task-irrelevant internal processes. This interpretation is consistent with the recently proposed hypothesis on beta-band functioning by Engel and Fries (2010), stressing beta’s relevance for maintaining ongoing motor activity and cognitive sets, particularly when the current state is prioritized over the processing of new signals, possibly by inhibiting new sensory input.

In this study, all participants were blindfolded, as it was part of a larger study investigating WM in congenital blindness (cf. section “Experimental Procedures”). Blindfolding changes the overall dynamic pattern of oscillatory brain activity (Berger, 1929; Adrian and Matthews, 1934; Geller et al., 2014). However, these general effects are constants; the reported load effects, thus, cannot be explained by blindfolding. Whether the overall topography of the observed WM effects at high load demands was modulated by blindfolding cannot be finally excluded but the parieto-occipital topography of attentional effects on alpha oscillations, as shown in a previous study (Wostmann et al., 2020), argues against this possibility.

## Conclusion

Taken together, our findings on WM maintenance-related activity in the EEG highlight that neural mechanisms that are recruited when individuals perform at their capacity limits were tendency altered after modality-specific WM training. This interesting finding, however, requires further research for confirmation. Additionally, replicating and extending previous WM load effects, we showed that successful WM maintenance at highly demanding load levels requires both a strengthening of task-relevant processing as well as an attenuation of task-irrelevant processing, characterized by specific electrophysiological signatures.

## Data Availability Statement

The datasets for this article are not publicly available because no consent for data sharing in repositories was given. Requests to access the datasets should be directed to HG-M helene.gudi-mindermann@posteo.de.

## Ethics Statement

The studies involving human participants were reviewed and approved by the the German Psychological Association (Deutsche Gesellschaft für Psychologie, DGPs). The participants provided their written informed consent to participate in the study.

## Author Contributions

All authors conceptualized the study and edited the manuscript. HG-M and JR wrote the manuscript. HG-M analyzed the data.

## Conflict of Interest

The authors declare that the research was conducted in the absence of any commercial or financial relationships that could be construed as a potential conflict of interest. The reviewer CB declared a shared affiliation, with no collaboration, with one of the authors, NK, to the handling Editor at the time of review.

## Footnotes

1 http://www.ru.nl/fcdonders/fieldtrip/

2 http://www.rstudio.com/

3 For example, during the adaptive *n*-back session one particular participant performed several blocks of 4-backs, 5-backs, 6-backs, and 7-backs. This participant’s 7-backs were coded as her/his individual maximal level (max), 6-backs as max–1, 5-backs as max–2, and 4-backs as max–3. Blocks with 4-back levels (max–3) were excluded from further analyses.
